# Factors associated with rapid and early virologic response to peginterferon alfa-2a/ribavirin treatment in HCV genotype 1 patients representative of the general chronic hepatitis C population

**DOI:** 10.1111/j.1365-2893.2009.01157.x

**Published:** 2010-02

**Authors:** M Rodriguez-Torres, M S Sulkowski, R T Chung, F M Hamzeh, D M Jensen

**Affiliations:** 1 Fundación de Investigación de DiegoSan Juan, Puerto Rico; 2 Johns Hopkins UniversityBaltimore, MD, USA; 3 Gastrointestinal Unit, Massachusetts General HospitalBoston, MA, USA; 4 Roche, NutleyNJ, USA; 5 University of ChicagoChicago, IL, USA

**Keywords:** chronic hepatitis C, on-treatment, pre-treatment, sustained virologic response

## Abstract

Rapid virologic response (RVR) and complete early virologic response (cEVR) are associated with sustained virologic response to hepatitis C virus (HCV) therapy. We retrospectively examined baseline and on-treatment factors associated with RVR (HCV RNA undetectable at week 4) and cEVR (HCV RNA undetectable at week 12, regardless of week 4 response). The analysis comprised 1550 HCV genotype-1 patients from five clinical trials, including three enriched with difficult-to-treat populations, randomized to peginterferon alfa-2a 180 μg/week plus ribavirin 1000–1200 mg/day. Overall, 15.6% achieved RVR and 54.0% achieved cEVR. Baseline factors predictive of RVR were serum HCV RNA ≤ 400 000 IU/mL (OR: 7.34; *P* < 0.0001), alanine aminotransferase >3 × ULN (OR: 2.01; *P* < 0.0001), non-cirrhotic status (OR: 1.92; *P* = 0.0087), age ≤ 40 years (OR: 1.56; *P* = 0.0085), white non-Latino ethnicity (OR: 1.41; *P* = 0.0666) and individual study (*P* < 0.0001). These factors plus body mass index ≤ 27 kg/m^2^ were predictive of cEVR. After adjusting for these factors, mean on-treatment ribavirin dose >13 mg/kg/day was predictive of RVR (OR: 1.69; *P* = 0.005) and cEVR (OR: 1.24; *P* = 0.09), whereas peginterferon alfa-2a dose reduction was not. Greater decreases in haematologic parameters were observed in patients who achieved cEVR compared with patients who did not. In conclusion, several baseline and on-treatment factors were associated with RVR and cEVR to peginterferon alfa-2a plus ribavirin in difficult-to-treat HCV genotype-1 patients, providing important prognostic information on the antiviral response in a patient cohort that is reflective of the general chronic hepatitis C population.

## Introduction

The recommended treatment for chronic hepatitis C (CHC) patients is combination therapy with pegylated interferon plus ribavirin. The goal of this treatment is a sustained virologic response (SVR), defined as the absence of detectable viral ribonucleic acid (RNA) at 6 months after the end of treatment. Despite improvements that have increased SVR rates, treating CHC remains a challenge in certain populations such as those patients infected with hepatitis C virus (HCV) genotype 1, with high viral load, cirrhosis or of African American race/ethnicity [1–3].

In a registration trial of peginterferon alfa-2a plus ribavirin, patients infected with HCV genotype 1 achieved an optimal SVR rate of 52% after 48 weeks of treatment [1]. Given the significant side-effects and healthcare costs associated with interferon therapies, identifying patients who are less likely to respond is highly desirable. Studies have demonstrated that early antiviral response is predictive of a SVR [4]. Patients who failed to achieve a complete early virologic response (cEVR) (defined as undetectable HCV RNA after 12 weeks of treatment) or a partial EVR (pEVR, defined as a ≥ 2-log_10_ decrease from baseline in HCV RNA after 12 weeks of treatment) had a lower likelihood of achieving SVR with an additional 36 weeks of treatment [2,5–7]. Rapid virologic response (RVR), defined as undetectable HCV RNA after 4 weeks of treatment [8], was found to be the single best predictor of SVR in HCV genotype 1-infected patients treated with peginterferon alfa-2a plus ribavirin [8].

Baseline factors that have been shown to predict SVR to peginterferon alfa-2a plus ribavirin include HCV genotype (other than type 1), age (≤40 years) and body weight (≤75 kg) [6]. Identifying factors that predict RVR and cEVR should further refine CHC treatment regimens. In patients infected with HCV genotype 1 and treated with peginterferon alfa-2a plus ribavirin for 24 weeks, baseline factors associated with RVR included lower viral load, absence of cirrhosis/bridging fibrosis and HCV genotype 1b [8]. Lower viral load was also associated with EVR/cEVR in patients treated with peginterferon alfa-2b plus ribavirin [9]. Recent evidence suggests that changes in haematologic parameters may be associated with virologic response to therapy [10]. Patients with a null response to peginterferon alfa-2a plus ribavirin demonstrated a smaller reduction in haematologic parameters than patients who achieved full virologic response, suggesting a systemic resistance to treatment [10].

Available data on the factors associated with achieving RVR and cEVR in difficult-to-treat HCV genotype 1-infected patients treated with a standard regimen, consisting of peginterferon alfa-2a 180 μg/week plus ribavirin 1000 or 1200 mg/day for 48 weeks, is limited. Identifying these factors may provide information to optimize and/or individualize the treatment of HCV genotype 1-infected patients, thus improving antiviral response. This is of particular importance because these difficult to treat populations have a high prevalence of HCV infection and comprise a large proportion of the HCV-infected population in the United States [11]. We, therefore, retrospectively analysed the data from five large clinical trials, three of which were enriched with difficult-to-treat patients, to examine the baseline and on-treatment factors associated with a RVR and cEVR to treatment with peginterferon alfa-2a plus ribavirin.

## Materials and Methods

### Patients

The analysis population consisted of all patients from five clinical trials who were infected with HCV genotype 1 (mono infection) and randomized to treatment for 48 weeks with subcutaneous peginterferon alfa-2a (Pegasys®; Roche, Nutley, NJ, USA; 180 μg/week) plus oral ribavirin (Copegus®; Roche, Nutley, NJ, USA; 1000 or 1200 mg/day) [1,3,6,12,13]. Three of these trials were enriched with difficult-to-treat patients, including one trial in which all 189 patients had high viral load (>800 000 IU/mL) at baseline (ClinicalTrials.gov Identifier: NCT00107653) [12]; one trial in which 78 of 106 patients (74%) were of black race [3]; and one trial in which 268 of 567 patients (47%) were of white Latino ethnicity (ClinicalTrials.gov Identifier: NCT00087607) [13].

### Study design

EVR was originally defined as cEVR (HCV RNA undetectable at week 12) or pEVR (HCV RNA detectable but >2 log decrease at week 12) regardless of week 4 RNA level. In this study, we investigated RVR (RNA undetectable at week 4) and cEVR (RNA undetectable at week 12, regardless of week 4 response). Patients with missing HCV RNA measurements at week 4 (or week 12), regardless of reason, were considered non-responders (non-RVR [or non-cEVR]) in the intent-to-treat (ITT) efficacy analyses of virologic end-points. There were small variations between studies in the detection limits for HCV RNA. Undetectable levels were defined as HCV RNA <28 IU/mL in one study (Roche High Pure System/COBAS® TaqMan® HCV Monitor Test) [14], <60 IU/mL in another study (Roche Amplicor polymerase chain reaction [PCR] assay) [12] and <50 IU/mL in the remaining three studies (Roche Amplicor PCR assay) [1,3,6].

### Statistical analysis

Baseline and demographic factors used in stepwise multiple logistic regression analyses included age (≤40 *vs* >40 years), sex, race/ethnicity (white non-Latino *vs* other), body mass index (BMI; ≤27 *vs* >27 kg/m^2^), baseline alanine aminotransferase (ALT) quotient (≤3 *vs* >3 × upper limit of normal [ULN]), serum HCV RNA concentration (≤400 000 *vs* >400 000 IU/mL), cirrhosis classification (cirrhotic *vs* non-cirrhotic) and individual study. The final model was based on the stepwise multiple variable logistic regression. Factors that had a *P* < 0.1 from the univariate analysis were allowed to enter the multiple regression model and factors with an adjusted *P* < 0.2 were kept in the model. Wald chi-square confidence intervals (CI) and *P*-values were used for testing the effects; *P* ≤ 0.2 indicated significant factors that remained in the final model. To account for differences between the studies, each individual study was forced into the model as a stratification factor regardless of its significance level. Odds ratios (OR) and 95% CI were calculated for the independent predictive factors.

Additional multiple variable logistic regression analyses included adjusting for all individually identified significant baseline prognostic factors and investigating the association of two on-treatment characteristics with RVR and cEVR: average daily exposure to ribavirin (≤13 *vs* >13 mg/kg) and peginterferon alfa-2a dose reductions (yes *vs* no).

Neutrophil counts, platelet counts and hemoglobin concentrations were assessed to determine if there were any associations between virologic and haematologic responses in these patients. Changes from baseline in these haematologic parameters were compared at weeks 4 and 12 in the RVR *vs* the non-RVR group, and in the cEVR *vs* the non-cEVR group. Haematologic changes result from the systemic effects of peginterferon and ribavirin and, therefore, can serve as indirect (surrogate) measures of host responses to interferons and may also reflect drug exposure and adherence.

## Results

### Baseline characteristics

**Fig. 1:**
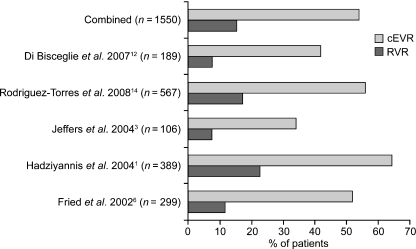
Percentage of patients with rapid virologic response (RVR) or complete early virologic response (cEVR) in individual studies of peginterferon alfa-2a plus ribavirin—intent-to-treat.

Patient demographics and characteristics at baseline are shown in Table 1. Of the 1550 patients treated with peginterferon alfa-2a plus ribavirin, 242 (15.6%) patients achieved RVR and 837 (54.0%) patients achieved cEVR (Table 1). The proportion of patients in the individual trials with a RVR ranged from 7.4% to 22.6%, while the proportion of patients with a cEVR ranged from 34.0% to 64.3% (Fig. 1). Patient baseline characteristics as a function of RVR and cEVR status and the *P*-values from the univariate analyses are presented in Table 2. In general, the patient characteristics that were more likely to be associated with achievement of RVR and cEVR were similar.

**Table 2:** Baseline characteristics and average daily ribavirin dose as a function of rapid virologic response (RVR) and complete early virologic response (cEVR)

	RVR		cEVR	
Characteristic	*n* (%)	*P*-value†	*n* (%)	*P*-value†
Number of patients*	242 (15.6)		837 (54.0)	
Sex				
Male	162 (15.7)	0.8659	549 (53.2)	0.4235
Female	80 (15.4)		288 (55.5)	
Age				
Age ≤40 years	100 (22.8)	<0.0001	298 (68.0)	<0.0001
Age >40 years	142 (12.8)		539 (48.5)	
Weight				
Weight ≤75 kg	104 (18.3)	0.0268	331 (58.4)	0.0095
Weight >75 kg	138 (14.0)		506 (51.5)	
BMI				
BMI ≤27 kg/m^2^	140 (19.1)	0.0009	435 (59.3)	0.0001
BMI >27 kg/m^2^	102 (12.8)		393 (49.3)	
Race				
White non-Latino	179 (17.1)	0.0268	611 (58.2)	<0.0001
White Latino	39 (13.2)		141 (47.6)	
Black	8 (5.2)		47 (30.5)	
Other	16 (31.4)		38 (74.5)	
ALT				
ALT quotient ≤3 × ULN	163 (13.7)	0.0002	612 (51.5)	0.0004
ALT quotient >3 × ULN	79 (21.8)		225 (62.2)	
HCV RNA				
Serum HCV RNA ≤400 000 IU/mL	125 (42.2)	<0.0001	222 (75.0)	<0.0001
Serum HCV RNA >400 000 IU/mL	117 (9.3)		615 (49.1)	
Cirrhotic classification				
Non-cirrhotic	217 (16.6)	0.0167	734 (56.0)	0.0002
Cirrhotic	25 (10.4)		103 (42.9)	
On-treatment average daily ribavirin dose				
≤13 mg/kg/day	50 (10.7)	0.0001	251 (47.7)	<0.0001
>13 mg/kg/day	192 (17.7)		586 (57.2)	

ALT, alanine aminotransferase; BMI, body mass index; ULN, upper limit normal.

* % Calculated relative to the total population (*n* = 1550).

† *P*-value from univariate analysis.

**Table 1:** Patient demographics, baseline characteristics and average daily ribavirin dose for the total population, rapid virologic response group (RVR) and complete early virologic response group (cEVR)

Characteristic	Total	RVR	cEVR
Number of patients, *n* (%)*	1550 (100.0)	242 (15.6)	837 (54.0)
Sex, *n* (%)†			
Male	1031 (66.5)	162 (66.9)	549 (65.6)
Age, years, mean ± SD	45.3 ± 9.24	42.2 ± 9.99	43.7 ± 9.31
Age ≤40 years, *n* (%)†	438 (28.3)	100 (41.3)	298 (35.6)
Weight, kg, mean ± sd	82.4 ± 18.1	78.2 ± 16.1	81.1 ± 17.8
Weight ≤75 kg, *n* (%)†	567 (36.6)	104 (43.0)	331 (39.5)
BMI, kg/m^2^, mean ± SD	28.0 ± 5.35	26.6 ± 4.46	27.5 ± 5.23
BMI ≤27 kg/m^2^, *n* (%)†	734 (47.4)	140 (57.9)	435 (52.0)
Race, *n* (%)†			
White non-Latino	1049 (67.7)	179 (74.0)	611 (73.0)
White Latino	296 (19.1)	39 (16.1)	141 (16.8)
Black	154 (9.9)	8 (3.3)	47 (5.6)
Other	51 (3.3)	16 (6.6)	38 (4.5)
ALT, *n* (%)†			
ALT quotient >3 × ULN	362 (23.4)	79 (32.6)	225 (26.9)
HCV RNA, log_10_, mean ± sd	6.2 ± 0.71	5.5 ± 0.92	6.1 ± 0.79
Serum HCV RNA			
≤400 000 IU/mL, *n* (%)†	296 (19.1)	125 (51.7)	222 (26.5)
≤800 000 IU/mL, *n* (%)	476 (30.7)	154 (32.4)	310 (65.1)
Cirrhotic classification, *n* (%)†			
Cirrhotic	240 (15.5)	25 (10.3)	103 (12.3)
On-treatment average daily ribavirin dose, *n* (%)†			
≤13 mg/kg	487 (31.4)	50 (20.7)	251 (30.0)
>13 mg/kg	1063 (68.6)	192 (79.3)	586 (70.0)

ALT, alanine aminotransferase; BMI, body mass index; SD, standard deviation; ULN, upper limit normal.

* % Calculated relative to the total population.

† % Calculated relative to RVR or cEVR groups.

### Patient disposition

Overall, a total of 16 patients (1%) and 40 patients (2.6%) withdrew from the study due to safety reasons by weeks 4 and 12, respectively. Seventeen patients (1.1%) and 44 patients (2.8%) withdrew due to non-safety reasons by weeks 4 and 12, respectively. Adverse events leading to withdrawal by week 4 included anaemia, chest pain, fatigue, rash, tongue oedema, headache, gastrointestinal events such as nausea and abdominal pain, and psychiatric effects such as aggression, suicide ideation and emotional instability.

### Multiple logistic regression analysis

The results of the multiple logistic regression analyses demonstrated that baseline factors independently associated with a RVR included serum HCV RNA ≤400 000 IU/mL (OR: 7.34; 95% CI 5.29–10.19; *P* < 0.0001), ALT quotient >3 × ULN (OR: 2.01; 95% CI 1.43–2.81; *P* < 0.0001), non-cirrhotic status (OR: 1.92; 95% CI 1.18–3.13; *P* = 0.0087), age ≤40 years (OR: 1.56; 95% CI 1.12–2.16; *P* = 0.0085), white non-Latino race (OR: 1.41; 95% CI 0.98–2.03; *P* = 0.0666) and individual study (*P* < 0.0001). The same independent factors were associated with a cEVR in this analysis: baseline serum HCV RNA ≤400 000 IU/mL (OR: 2.81; 95% CI 2.07–3.81; *P* < 0.0001), non-cirrhotic status at baseline (OR: 1.95; 95% CI 1.43–2.65; *P* < 0.0001), age ≤40 years (OR: 1.81; 95% CI 1.41–2.34; *P* < 0.0001), baseline ALT quotient >3 × ULN (OR: 1.61; 95% CI 1.24–2.09; *P* = 0.0003), white non-Latino race (OR: 1.59; 95% CI 1.24–2.04; *P* = 0.0002) and individual study (*P* < 0.0001). In addition, BMI (≤27 *vs* >27 kg/m^2^) was associated with a cEVR in this analysis (OR: 1.21; 95% CI 0.97–1.50; *P* = 0.0925). In this report we defined HCV RNA <400 000 IU/mL as low viral load. However, similar results were obtained when the analysis was repeated with higher HCV RNA cut-off levels (≤600 000 *vs* >60 000 IU/mL and ≤800 000 *vs* >800 000 IU/mL). Also, similar results were also obtained when the analysis was repeated using different BMI cut-off levels (>25 *vs*≤25 kg/m^2^ and >30 *vs*≤30 kg/m^2^) [M. Rodriguez-Torres, L.J. Jeffers, M.Y. Sheikh, L. Rossaro, V.A. Sey, F.M. Hamzeh and P. Martin, unpublished data].

After adjusting for significant risk factors in the baseline characteristics model (Table 3), multiple logistic regression analysis showed that compared with an average on-treatment daily ribavirin dose of ≤13 mg/kg, an average on-treatment daily ribavirin dose of >13 mg/kg was a predictor of RVR (OR: 1.69; 95% CI 1.17–2.43; *P* = 0.0049) and cEVR (OR: 1.24; 95% CI 0.97–1.59; *P* = 0.0907). BMI was associated with cEVR before, but was not after, adjusting for ribavirin exposure. BMI was not associated with RVR (Table 3). In contrast, peginterferon alfa-2a dose reduction (yes *vs* no) was not a predictor of RVR or cEVR.

**Table 3:** Summary of multiple logistic regression analysis for baseline factors and average daily ribavirin dose predictive of rapid virologic response (RVR) and complete early virologic response (cEVR)adjusting for significant baseline prognostic factors

	RVR		cEVR	
Factor	OR (95% CI)	*P*-value*	OR (95% CI)	*P*-value*
Race				
White non-Latino *vs* other	1.45 (1.00–2.08)	0.0474	1.61 (1.25–2.06)	0.0002
Age				
≤40 *vs* >40 years	1.51 (1.08–2.09)	0.0145	1.81 (1.40–2.32)	<0.0001
Baseline ALT quotient				
>3 *vs*≤3 × ULN	2.06 (1.47–2.89)	<0.0001	1.61 (1.24–2.10)	0.0003
Baseline serum HCV RNA				
≤400 000 *vs* >400 000 IU/mL	7.25 (5.23–10.07)	<0.0001	2.81 (2.07–3.82)	<0.0001
Cirrhosis				
Non-cirrhotic *vs* cirrhotic	1.94 (1.19–3.17)	0.0077	1.96 (1.44–2.68)	<0.0001
Individual study		<0.0001		<0.0001
Average daily ribavirin dose†				
>13 mg/kg *vs*≤13 mg/kg)	1.69 (1.17–2.43)	0.0049	1.24 (0.97–1.59)	0.0907

ALT, alanine aminotransferase; CI, confidence intervals; OR, odds ratios; ULN, upper limit normal.

* A significance level of *P* < 0.1 was used to qualify entrance into the multiple logistic regression model, and factors with *P* ≤ 0.2 were allowed to stay in the final model. Study was forced into the model, regardless of its significance.

† On-treatment average daily ribavirin dose.

### Haematologic effects of peginterferon plus ribavirin

The association of other host pharmacodynamic effects of peginterferon and ribavirin with antiviral activity was investigated (Table 4). In the RVR and non-RVR groups, mean changes in neutrophil counts at weeks 4 and 12 decreased from baseline to a similar extent with no difference observed between the groups. In the cEVR and non-cEVR groups, mean changes in neutrophil counts at weeks 4 and 12 also decreased from baseline; however, the cEVR group had a greater decrease in neutrophil counts at week 12 than the non-cEVR group (*P* = 0.0003). Platelet counts decreased from baseline at weeks 4 and 12 in all groups although the changes were not significant. Mean haemoglobin concentrations also decreased between weeks 4 and 12 in all groups. The decrease from baseline to week 12 in the cEVR group was greater than that in the non-cEVR group (*P* = 0.0160). In the univariate analysis, body weight and on-treatment drug exposure were both associated with RVR and cEVR. The influence of these factors on the observed decreases in haematologic parameters between the response and non-response groups was analysed. After adjusting for body weight and on-treatment drug exposure, the decrease from baseline to week 12 in neutrophil counts and haemoglobin concentrations in the cEVR group compared with the non-cEVR group remained significant (*P* = 0.0008 and *P* = 0.0179, respectively); the decrease in platelet counts remained non-significant (*P* = 0.2093) [M. Rodriguez-Torres, L.J. Jeffers, M.Y. Sheikh, L. Rossaro, V.A. Sey, F.M. Hamzeh and P. Martin, unpublished data]. The results in the RVR and non-RVR groups also remained unchanged. This suggests that body weight and on-treatment drug exposure were not confounding factors in the observed haematologic changes between the response and non-response groups.

**Table 4:** Changes in neutrophil counts, platelet counts and haemoglobin concentration at weeks 4 and 12 in patients treated with peginterferon alfa-2a plus ribavirin

	RVR		Non-RVR			cEVR		Non-cEVR		
	*n*	Change from baseline	*n*	Change from baseline	*P*-value*	*n*	Change from baseline	*n*	Change from baseline	*P*-value†
Neutrophil count‡										
Week 4	228	−1.8 ± 1.35	1246	−2.0 ± 1.39	–	801	−2.1 ± 1.38	673	−1.8 ± 1.38	–
Week 12	230	−1.9 ± 1.77	1236	−2.0 ± 1.65	0.7969	826	−2.2 ± 1.52	640	−1.8 ± 1.82	0.0003
Platelet count‡										
Week 4	233	−56.7 ± 43.80	1243	−47.2 ± 46.90	–	805	−53.5 ± 46.75	671	−43.0 ± 45.68	–
Week 12	231	−66.4 ± 46.80	1234	−66.1 ± 49.73	0.4846	824	−70.7 ± 50.74	641	−60.2 ± 46.68	0.0859
Haemoglobin concentration§										
Week 4	241	−2.6 ± 1.80	1300	−2.7 ± 1.94	–	836	−2.6 ± 1.86	705	−2.8 ± 1.99	–
Week 12	239	−3.2 ± 1.74	1279	−3.2 ± 1.84	0.3827	835	−3.2 ± 1.71	683	−3.2 ± 1.96	0.0160

ANCOVA, analysis of covariance; cEVR, complete early virologic response; RVR, rapid virologic response; SD, standard deviation. Level of statistical significance for haematologic analysis set at 0.05.

* *P*-value for between RVR and non-RVR group change from baseline at week 12 based on ANCOVA with baseline values and responder group as covariates.

† *P*-value for between cEVR and non-cEVR group change from baseline at week 12 based on ANCOVA with baseline values and responder group as covariates.

‡ ×10^3^/μL, mean ± SD.

§ g/dL, mean ± sd.

## Discussion

The response to antiviral therapy in HCV-infected patients is heterogeneous and, despite increases in SVR rates, treatment outcomes with peginterferon alfa-2a plus ribavirin are not optimal in certain patient populations and might still be improved [1,2]. Monitoring the early antiviral response to therapy can help identify those patients who are less likely to achieve SVR and therefore provide critical information for the overall management of patients with CHC.

Both RVR and cEVR are associated with the achievement of SVR in patients with CHC [15]. Identifying the baseline and on-treatment factors, both virus and patient related, that are associated with RVR and cEVR to therapy with peginterferon alfa-2a plus ribavirin may help predict a patient’s response to treatment, as well as provide important information that can be used to improve viral outcomes [15–17]. For example, the modification of baseline and/or on-treatment factors, such as drug dose, may help increase the likelihood of successful response to therapy. Furthermore, early identification of those patients who are unlikely to achieve RVR or cEVR may help to reduce unnecessary healthcare expenses and limit the side-effects associated with drug exposure.

The analysis presented here demonstrated that several patient characteristics, including lower serum HCV RNA, higher ALT quotient, absence of cirrhosis, younger age and white non-Latino race/ethnicity were associated with successful achievement of RVR and cEVR in patients infected with HCV genotype 1. In addition, lower BMI was associated with achieving cEVR. The effects of peginterferon-alfa 2a dose and ribavirin dose on RVR and cEVR were also analysed. Ribavirin dose was a predictor of RVR and cEVR, whereas peginterferon-alfa 2a dose or dose reduction was not. This finding is in agreement with previous studies that evaluated the effect of ribavirin dose reductions on virologic response rates in HCV genotype 1-infected patients [18]. Our analysis showed that, when combined with peginterferon alfa-2a 180 μg/week, higher daily on-treatment ribavirin exposure increased the likelihood of achieving RVR and cEVR, even in difficult-to-treat patients infected with HCV genotype 1. Specifically, ribavirin exposure >13 *vs*≤13 mg/kg/day was associated with a greater proportion of patients achieving RVR and cEVR (17.7%*vs* 10.7% and 57.2%*vs* 47.7%, respectively). Further analysis to determine the effect of average on-treatment daily ribavirin dose on the likelihood of RVR and cEVR suggested that a linear relationship existed up to ∼12 and 10 mg/kg/day, respectively, after which the effect remained relatively constant (data not shown). However, cut-off points could not be identified since no clear inflection points were obtained. Individual study was also found to be predictive of RVR and cEVR even after adjusting for the effects of known factors such as Latino ethnicity which accounted for ∼50% of the LATINO study population [13]. Beyond the known factors (such as black or Latino ethnicity or having a large proportion of patients with a high baseline viral load), other unknown factors (such as differences between studies in time effects or improvements in HCV treatment practices over time) and variables such as different peginterferon and ribavirin dose adjustment protocols may have contributed to individual study being a predictive factor.

Limited data have been published regarding the factors associated with RVR and cEVR. A few studies have shown that in patients receiving peginterferon alfa plus ribavirin, lower viral load, younger age, lower body weight and absence of advanced fibrosis may be associated with a RVR [8,19–21]. Jensen *et al.* [8] conducted a *post hoc* analysis of 216 HCV genotype 1-infected patients randomized to 24 weeks of treatment with peginterferon alfa-2a 180 μg/week plus ribavirin either 800 mg/day (low dose) or 1000/1200 mg/day (standard dose), and showed that baseline viral load was a significant predictor of RVR. Although the overall RVR rate was 23.6%, patients with baseline HCV RNA <200 000 IU/mL, 200 000–600 000 IU/mL and >600 000 IU/mL had RVR rates of 49.1%, 26.0% and 9.2%, respectively. Statistical analysis showed that, relative to patients with baseline HCV RNA >600 000 IU/mL, those patients with HCV RNA <200 000 IU/mL (OR: 9.7; 95% CI 4.2–22.5; *P* < 0.0001) or 200 000–600 000 IU/mL (OR: 3.6; 95% CI 1.5–9.1; *P* = 0.0057) were significantly more likely to achieve RVR. Overall, SVR rates were considerably higher in patients who achieved RVR than in those patients who did not (89%*vs* 19%), and RVR was a significant predictor of SVR (OR: 23.7; 95% CI 9.1–61.7; *P* < 0.0001). In addition, patients infected with HCV subtype 1b were more likely to achieve RVR than those patients infected with HCV subtype 1a (OR: 1.8; 95% CI 0.9–3.7; *P* = 0.095). A lack of RVR was also associated with liver fibrosis or cirrhosis. More recently, in a multicenter study conducted in the south of Italy, 696 HCV genotype 1-infected patients were randomized to treatment with peginterferon alfa-2a or alfa-2b, each plus ribavirin, for either 48 weeks or for an individualized duration based on the time at which HCV RNA undetectability was first achieved [20]. In univariate analysis, young age (*P* = 0.004), low baseline viral load (*P* = 0.0001) and fibrosis stage ≤2 (*P* = 0.0001) were associated with RVR. In multivariate analysis, HCV RNA levels <400 000 IU/mL (OR: 2.27; 95% CI 1.49–3.41) and absence of advanced fibrosis (OR: 1.40; 95% CI 1.15–1.64) were independently associated with achieving a RVR. Lower baseline viral load (<400 000 IU/mL) was also the only significant factor associated with a RVR (OR: 3.052; 95% CI 1.706–5.458) in 200 previously untreated Taiwanese patients infected with HCV genotype 1 who were randomized to treatment with peginterferon alfa-2a plus ribavirin for either 24 or 48 weeks [21]. While our results are in agreement with these studies, we believe the analysis reported here is the first to include an ethnically diverse cohort that is representative of the general CHC population.

Haematologic adverse effects are relatively common when interferons are combined with ribavirin [22] and can also reflect a patient’s response to therapy. In patients receiving peginterferon/ribavirin, haematologic changes can be considered a marker of pharmacodynamic effects, and may correlate with anti-HCV activities. Similarly, a lack of a haematologic effect may indicate a poor response to therapy. Indeed, it has recently been reported that null virologic response to combination therapy is associated with a blunted haematologic response, suggesting null responders are resistant to interferon therapy [10]. In our analysis, neutrophil counts, platelet counts and haemoglobin concentrations decreased from baseline to weeks 4 and 12 in all groups. While there was no difference in haematologic response between the RVR and non-RVR groups, significantly greater decreases in haematologic parameters at week 12 were shown in patients who achieved cEVR when compared with those patients who did not achieve cEVR. The absence of any observable difference in haematologic side-effects between the RVR and non-RVR groups may be due to the smaller proportion of patients achieving RVR. Alternatively, it could be due to the nadir of haematologic adverse events occurring later than 4 weeks (6–8 weeks) [12]. In addition, the body’s ability to mount a haematologic proliferation response may have been able to compensate during the early phase of therapy, thus masking the pharmacodynamic effects of the treatment. The observed blunted response to combination therapy in the non-cEVR group suggests that patient factors may be partly responsible for the individual variability in the systemic response to peginterferon and/or ribavirin. Analysis of these patient factors may prove useful in improving treatment outcomes, as well as further our understanding of the pharmacokinetic/pharmacodynamic relationship between drug dose and antiviral response. For example, patients who required dose reductions may be more likely to be responders than non-responders, thus suggesting that antiviral response and haematologic effects are, in essence, the pharmacodynamic effects of interferons and ribavirin. Furthermore, it may also reflect a different pharmacokinetic profile in these patients, as well as indirectly correlate with adherence and total exposure [23,24].

In the current analysis, 16% of patients achieved RVR. This rate is lower than the rates reported in other studies [8,20,21] and is likely due to the number of studies in this analysis that were enriched with patient populations that are considered more difficult to treat. For example, patients with viral loads >400 000 and >800 000 IU/mL comprised ∼81% and 70% of the total study population, respectively; black patients and white Latino patients comprised ∼30%, which is reflective of the general CHC population. Furthermore, the heterogeneous characteristics of the patients among the five studies included in this analysis are also likely to contribute to the range of RVR rates observed (7–23%). In fact, in the two trials with the lowest RVR rates, the majority of patients were black [3] or had a high baseline viral load (>800 000 IU/mL) [12]. In contrast, the study with the highest RVR rate had a relatively small proportion of patients with poor prognostic markers [1]. Thus, by including a diverse range of populations that more closely represents the prevalence distribution of HCV infection in the United States, our analysis provides a meaningful assessment of the virologic response rates to treatment with peginterferon alfa-2a plus ribavirin, and underscores the need to develop more effective treatment strategies for these patients.

In this retrospective analysis, we demonstrated that baseline and on-treatment factors, including lower serum HCV RNA concentration, higher ALT quotient, absence of cirrhosis, younger age, white non-Latino ethnicity and daily on-treatment ribavirin dose >13 mg/kg, were independently associated with achieving RVR and cEVR in patients infected with HCV genotype 1 and treated with peginterferon alfa-2a plus ribavirin for 48 weeks. There is a need to improve further the efficacy of combination therapy, as well as limit the associated side-effects and healthcare costs, especially in those patient populations considered difficult to treat. Optimizing the antiviral response is crucial for accomplishing these goals. Since ribavirin dose is one of the only modifiable factors identified in this analysis that is associated with an increase in RVR and cEVR, physicians may be advised to maintain a higher ribavirin dose to increase the likelihood of achieving SVR. Thus, identifying the predictors of response to peginterferon alfa-2a plus ribavirin therapy and tailoring treatment regimens for individual patients based on their risk profile may be one approach for achieving maximum antiviral response. These findings will need to be supported by large, prospective clinical studies that are designed to evaluate whether SVR rates can be increased by modifying baseline or on-treatment factors.

## Abbreviations:

ALT: alanine aminotransferase
BMI: body mass index
cEVR: complete early virologic response
CHC: chronic hepatitis C
CI: confidence interval
EVR: early virologic response
HCV: hepatitis C virus
ITT: intent-to-treat
OR: odds ratio
RNA: ribonucleic acid
RVR: rapid virologic response
SVR: sustained virologic response
ULN: upper limit of normal
